# Indoleamine 2,3-dioxygenase 1 (IDO1) activity correlates with immune system abnormalities in multiple myeloma

**DOI:** 10.1186/1479-5876-10-247

**Published:** 2012-12-11

**Authors:** Giuseppina Bonanno, Andrea Mariotti, Annabella Procoli, Valentina Folgiero, Daniela Natale, Luca De Rosa, Ignazio Majolino, Linda Novarese, Alberto Rocci, Manuela Gambella, Marilena Ciciarello, Giovanni Scambia, Antonio Palumbo, Franco Locatelli, Raimondo De Cristofaro, Sergio Rutella

**Affiliations:** 1 Department of Gynecology, Catholic University Med. School, Rome, Italy; 2 Department of Pediatric Hematology/Oncology, IRCCS Bambino Gesù Children’s Hospital, Rome, Italy; 3 Department of Hematology, Azienda Ospedaliera “S. Camillo-Forlanini”, Rome, Italy; 4 Department of Medicine, Hemostasis Research Centre, Catholic University Med. School, Rome, Italy; 5 Myeloma Unit, Division of Hematology, University of Turin, AOU S. Giovanni Battista, Turin, Italy; 6 Institute of Hematology and Medical Oncology “L.&A. Seràgnoli”, University of Bologna, Bologna, Italy; 7 University of Pavia, Pavia, Italy

## Abstract

**Background:**

Multiple myeloma (MM) is a plasma cell malignancy with a multifaceted immune dysfunction. Indoleamine 2,3-dioxygenase 1 (IDO1) degrades tryptophan into kynurenine (KYN), which inhibits effector T cells and promote regulatory T-cell (Treg) differentiation. It is presently unknown whether MM cells express IDO1 and whether IDO1 activity correlates with immune system impairment.

**Methods:**

We investigated IDO1 expression in 25 consecutive patients with symptomatic MM and in 7 patients with either monoclonal gammopathy of unknown significance (MGUS; n=3) or smoldering MM (SMM; n=4). IDO1-driven tryptophan breakdown was correlated with the release of hepatocyte growth factor (HGF) and with the frequency of Treg cells and NY-ESO-1-specific CD8^+^ T cells.

**Results:**

KYN was increased in 75% of patients with symptomatic MM and correlated with the expansion of CD4^+^CD25^+^FoxP3^+^ Treg cells and the contraction of NY-ESO-1-specific CD8^+^ T cells. *In vitro*, primary MM cells promoted the differentiation of allogeneic CD4^+^ T cells into *bona fide* CD4^+^CD25^hi^FoxP3^hi^ Treg cells and suppressed IFN-γ/IL-2 secretion, while preserving IL-4 and IL-10 production. Both Treg expansion and inhibition of Th1 differentiation by MM cells were reverted, at least in part, by d,l-1-methyl-tryptophan, a chemical inhibitor of IDO. Notably, HGF levels were higher within the BM microenvironment of patients with IDO^+^ myeloma disease compared with patients having IDO^-^ MM. Mechanistically, the antagonism of MET receptor for HGF with SU11274, a MET inhibitor, prevented HGF-induced AKT phosphorylation in MM cells and translated into reduced IDO protein levels and functional activity.

**Conclusions:**

These data suggest that IDO1 expression may contribute to immune suppression in patients with MM and possibly other HGF-producing cancers.

## Background

The establishment of anti-tumor immunity requires the interaction of different cell types including, among others, APC and T cells. Escape from immunosurveillance through immunoselection, also known as immunoediting, and immunosubversion, i.e., active suppression of the immune response, is a hallmark of cancer [1]. In this respect, naturally occurring CD4^+^FoxP3^+^ regulatory T cells (Treg), a T-cell subset frequently over-represented in cancer-bearing hosts, were shown to suppress tumor-associated antigen (TAA)-reactive T cells, both *in vitro*[2] and *in vivo*[3]. Indoleamine 2,3-dioxygenase 1 (IDO1) is a tryptophan-catabolizing enzyme encoded by the *IDO1* gene. IDO1 oxidizes tryptophan into *N*-formylkynurenine, which is rapidly converted to kynurenine (KYN) by the action of KYN formamidase [4]. The same reaction can be catalyzed by tryptophan 2,3-dioxygenase (TDO), a heme-containing cytosolic enzyme encoded by *TDO2* gene and detected at high levels in the liver [5]. In humans, IDO1 is expressed by a unique subset of dendritic cells (DC) [6], by acute myeloid leukemia [7,8] and by a variety of solid tumors, such as colorectal cancer [9], melanoma [10] and serous ovarian cancer [11]. The IDO1-driven production of KYN promotes the development, stabilization and activation of Treg cells, while suppressing effector T cells, all of which may contribute to immune system impairment in cancer-bearing individuals [12]. Recently, a mechanism of tumoral immune resistance centered on tryptophan degradation by TDO has been described in human tumors, such as melanoma, hepatocarcinoma, glioma and bladder carcinoma, but not in leukemia or lymphoma [13,14]. Multiple myeloma (MM) is a malignant plasma cell (PC) disorder, accounting for approximately 1% of neoplastic diseases and 13% of hematological cancers [15], and evolving from a monoclonal gammopathy of undetermined significance (MGUS) that progresses to smoldering myeloma (SMM) and, finally, to symptomatic MM. In recent years, the introduction of autologous hematopoietic stem cell transplantation (HSCT) and the availability of novel drugs such as thalidomide, lenalidomide and bortezomib, have prolonged overall survival [16,17]. Importantly, MM tumor cells are susceptible to immune recognition in the form of graft-versus-myeloma effect, as suggested by the therapeutic efficacy of allogeneic HSCT. Indeed, in 162 cases of newly diagnosed MM, event-free and overall survival were improved in patients given autologous-allogeneic HSCT (tandem transplantation) as compared with patients lacking an HLA-matched sibling donor and receiving double autologous HSCT [18].

MM is unique in its ability to elude immunosurveillance, as a result of qualitative and/or quantitative abnormalities of DC and Treg cells [19], and of enhanced release of immunoregulatory cytokines by microenvironmental cells [20]. For instance, interaction between myeloma cells and plasmacytoid DC in MM bone marrow (BM) triggers the release of known MM-cell growth factors, including IL-10, IL-6, and MCP-1 or IP10 [21]. Furthermore, BM stromal cells (BMSC) in MM secrete immunomodulatory and pro-angiogenic molecules, such as TGF-β, vascular endothelial growth factor, IL-6 and hepatocyte growth factor (HGF) [22,23]. HGF is a 90-kd protein that signals through the MET receptor and is endowed with previously unappreciated effects on the immune response, as shown both *in vitro* and *in vivo*[24,25]. HGF confers tolerogenic functions to human, monocyte-derived (Mo)-DC by up-regulating IDO1 expression and IL-10 secretion [26]. Several MM cell lines both release and activate HGF by secreting HGF-activator (HGFA), a factor XIIa-related serine protease [27]. Importantly, high levels of HGF in serum and BM fluid of patients with MM predict a dismal prognosis, with a survival time of 32 and 21 months for patients with low and high HGF, respectively [23,28]. HGF values have been reported to decline after treatment with high-dose chemotherapy in patients with MM who obtain at least a partial response [29]. The expression and function of IDO1 in MM have not been investigated previously. We provide evidence that KYN are increased in the peripheral blood (PB) and BM of patients with MM, in correlation with HGF release, expansion of Treg cells and shrinkage of NY-ESO-1-specific CD8^+^ T cells. *In vitro*, HGF antagonism with SU11274, a chemical inhibitor of MET, translated into the down-regulation of IDO protein and functional activity in MM cells.

## Methods

### Abs and reagents

The following tumor cell lines were obtained from American Type Culture Collection (ATCC; LGC Standards, Milan, Italy): U266, MOLP-8, RPMI-8226 (human MM) and U87-MG (human glioma). Primary rat hepatocytes were a generous gift of Anna Alisi (OPBG, Rome). CD138 Microbeads, Human CD4^+^CD25^+^ Regulatory T-Cell Isolation Kit, Monocyte Isolation Kit II, Treg Suppression Inspector, Mo-DC Differentiation Medium, Mo-DC Maturation Medium and anti-CD166 (ALCAM or activated leukocyte cell adhesion molecule) mAb (3A6 clone) were purchased from Miltenyi Biotec (Bergisch Gladbach, Germany). Anti-human IDO antibodies (clone 700838), recombinant human IFN-γ, IL-2 and HGF, as well as ELISA kits for the quantification of IL-10, TGF-β1, IFN-γ and HGF, were all purchased from R&D Systems (Oxon, Cambridge, UK). The IDO chemical inhibitor d,l-1MT, a racemic mixture containing both the *levo* and the *dextro* isomer of 1MT, L-tryptophan, KYN, PMA and ionomycin were obtained from Sigma Chemicals (St. Louis, MO). 6-Fluoro-3-[(1*E*)-2-(3-pyridinyl)ethenyl]-1*H*-indole (680C91), a selective and potent TDO inhibitor (Tocris Bioscience; Bristol, UK) was used at 5 μM final concentration [5,14]. STAT3 Inhibitor III (WP1066) was purchased from Santa Cruz Biotechnology (Milan, Italy). The Human FoxP3 Staining Set was purchased from eBioscience (San Diego, CA). FITC-conjugated anti-CD8, FITC-conjugated anti-HLA-A2, PerCP-conjugated anti-CD19 mAb, PE-conjugated anti-IL-2 mAb, anti-IL-4 and IL-10 mAb, FITC-conjugated anti-IFN-γ and anti-IL-17 mAb, FITC-conjugated anti-CD14 mAb, PE-conjugated anti-CD1a mAb, Cytofix/Cytoperm™ solution and Golgi Plug Protein Transport Inhibitor™ were purchased from BD Biosciences (Mountain View, CA). PE-labeled NY-ESO-1_157-165_ pentamers (peptide sequence: SLLMWITQV) and PE-labeled influenza A_58-66_ pentamers (peptide sequence: GILGFVFTL) were obtained from Pro Immune (Oxford, UK). Rabbit anti-human antibodies to Akt (pan) (C67E7) and phosphorylated Akt (Ser473) (D9E) were purchased from Cell Signaling Technology (Milan, Italy). Rabbit anti-human IDO (H-101 clone) and anti-TDO2 antibodies (N1C1 clone) were purchased from Santa Cruz Biotechnology and from GeneTex (Irvine, CA), respectively. Mouse anti-human NY-ESO-1 antibodies were obtained from Life Technologies (Milan, Italy). The Cell Fixation and Permeabilization Kit was obtained from Invitrogen (Milan, Italy). Mouse anti-human GAPDH antibodies were from Millipore (Milan, Italy). Isotypic control Abs were purchased from BD Biosciences and eBioscience, as appropriate. SU11274, a selective MET inhibitor [30], was purchased from Calbiochem (La Jolla, CA).

### Patients’ characteristics

Twenty-five consecutive patients with MM and 7 patients with either MGUS (n=3) or SMM (n=4) participated into the study (Table 1), which was approved by the local Ethical Committee (protocol #P/53/CE/2010). Surplus diagnostic material, i.e., PB and BM samples collected on Hospital admission (disease onset) or during follow-up visits, was used for the cellular and molecular studies detailed below, after obtaining patients’ informed consent. The International Staging System (ISS) was used to classify myeloma disease [31]. Patients with ISS stages II and III were grouped together in the analysis, as they are considered to have higher-risk disease [32].

**Table 1:** Patients’ characteristics

**UPN**	**Diagnosis**	**Disease status**	**Light chain isotype**	**Cytogenetics**	**ISS stage**	**Therapy at sampling**	Clinical status*
1	MM	Relapse	IgG κ	Normal	III	PDN, Cy	Deceased
2	SMM	Onset	IgG λ	t(4;14)	N.A.	None	Alive/SMM
3	MM	Stable disease	Non secretory	Normal	I	None	Alive/PR
4	SMM	Onset	IgA κ	Normal	N.A.	None	Alive/SMM
5	MM	Partial response	IgG κ	Normal	I	PDN, Thalidomide	Alive/PR
6	MM	Onset	IgG κ	Del(13q); t(4;14)	I	None	Deceased
7	MM	Onset	IgA λ	Normal	III	None	Lost at follow-up
8	MM	Onset	IgG λ	Del(13q)	I	None	Alive/PR
9	MM	Onset	IgD λ	Del(13q)	I	VMPT	Alive/CR
10	MGUS	--	Micromolecular, λ	Normal	N.A.	None	Alive/MGUS
11	MM	Onset	Micromolecular, κ	Normal	I	Prednisone	Alive/Stable disease
12	MGUS	--	IgG κ	Normal	N.A.	None	Alive/MGUS
13	MM	Onset	Micromolecular, κ	Normal	III	Auto-HSCT	Alive/CR
14	MM	Relapse	IgA κ	Del(13q)	I	Prednisone	Alive/PD
15	MM	Onset	NA	Normal	II	None	NA
16	MM	Partial response	IgG λ	Normal	III	PDN	Alive/Stable disease
17	MM	Relapse	IgA λ	Normal	III	PDN	Alive/PR
18	SMM	Onset	IgA κ	Normal	N.A.	None	Alive/progressed to MM
19	MM	Relapse	IgA κ	p53 mutation	II	None	Alive/PR
20	MM	Stable disease	IgG κ	Normal	I	MP	Deceased
21	SMM	Onset	IgG λ	Normal	N.A.	None	Alive/SMM
22	MGUS	--	IgG κ	Normal	N.A.	None	Alive/MGUS
23	MM	Onset	IgG κ	t(11;14);14q32 translocation	I	None	Alive
24	MM	Onset	IgG κ	Del(13q); del(17p)	II	None	Alive
25	MM	Relapse	IgA	Del(13q); t(4:14)	II	None	Alive, relapse
26	MM	Onset	Micromolecular, λ	ND	III	None	Alive/PR
27	MM	Relapse	Non secretory	ND	I	None	Alive/VGPR
28	MM	Relapse	IgA λ	Normal	I	None	Alive/VGPR
29	MM	Onset	IgA κ	Del(13q); del(17p)	III	None	Alive/PD
30	MM	Onset	IgA λ	Del(13q); t(4:14)	I	None	Alive/PR
31	MM	Relapse	IgG κ	ND	I	Prednisone	Alive/PD
32	MM	Relapse	IgG κ	ND	I	None	Alive/PR

Patients’ characteristics. Patients’ demographics, disease features, medical therapies and disease status at sampling are shown. Abbreviations: *SMM* smoldering multiple myeloma, *CR* complete remission, *PR* partial remission, *PD* progressive disease, *VGPR* very good partial response, *NA* not applicable, *ND* not done, *BJ* Bence-Jones proteinuria. *PDN* prednisone, *Cy* cyclophosphamide, *MP* melphalan + prednisone, *VMPT* bortezomib, melphalan, prednisone and thalidomide

*12 months after inclusion in the study.

### Cell and serum preparation

PB and BM samples collected at simultaneous time-points were used to isolate PBMC and BMMC by density gradient centrifugation on Ficoll-Hypaque (Uppsala, Sweden). Cells were either used fresh or were stored in FCS with 10% dimethyl sulfoxide in the vapor phase of liquid nitrogen until the day of experimental manipulation. Sera obtained by centrifugation of clotted PB and BM samples at 400g for 20 minutes were rapidly frozen and stored at −80°C until analysis. Primary MM cells were purified by positive selection with CD138 microbeads, according to the manufacturer’s instructions. Blood samples were also obtained by consented age- and sex-matched healthy blood donors and were used for the measurement of KYN, tryptophan and HGF as well as for the enumeration of FoxP3-expressing Treg cells.

### Expansion of BMSC

BMMC were cultured in RPMI 1640 supplemented with 20% FCS to establish BMSC [33]. The percentage of BMSC was evaluated after labeling with an anti-CD166 mAb. After 3 weeks in culture, BMSC were either challenged with 100 IU/ml IFN-γ for 24 hours or were left untreated. Culture supernatants were collected at baseline and after IFN-γ provision to measure tryptophan levels and KYN production, as will be detailed.

### Mixed tumor-cell lymphocyte cultures (MTLC)

CD25^+^ cells were positively selected from buffy-coat preparations of consenting blood donors with directly conjugated anti-CD25 magnetic microbeads (4 μl per 10^7^ cells) [26]. The remaining non-CD25^+^ fraction was used to isolate CD4^+^CD25^-^ cells by positive selection with anti-CD4 mAb-coated microbeads. Primary MM cells were cultured under serum-free conditions (10% BIT HCC-9500; Stem Cell Technologies, Vancouver, BC) with allogeneic CD4^+^ T cells in RPMI 1640 medium [26,34]. After 6 days, cells were harvested to evaluate cytokine secretion and expression of Treg-associated markers. In selected wells, IL-2 was provided to the MTLC at 10 IU/ml. The IDO chemical inhibitor d,l-1MT was used at 200 μM to assess the role of IDO1 in the conversion of Treg cells by MM cells.

### Cell proliferation tracking

Freshly isolated CD4^+^ T cells were labeled with CFSE (2.5 μM; Molecular Probes, Eugene, OR) for 10 minutes at room temperature. After washings in PBS supplemented with 3% FCS, cells were used for co-culture experiments. The analysis of CFSE dilution in the proliferating cell progeny was pursued with the Mod Fit® LT 2.0 software (Verity Software House Inc., Topsham, ME) [35].

### Treg suppression assays

Treg cells emerging from the MTLC were purified as above described and were co-cultured with allogeneic CD4^+^CD25^-^ responder T cells that were pre-loaded with CFSE. T-cell proliferation was induced with an optimized polyclonal stimulus, consisting of anti-CD2+anti-CD3+anti-CD28 mAb.

### Quantification of Treg cells and NY-ESO-1-specific CD8^+^ T cells by flow cytometry

The percentage of CD4^+^CD25^+^FoxP3^+^ Treg cells was estimated as previously published [26]. Appropriate fluorochrome-conjugated, isotype-matched mAb helped establish background fluorescence. Treg cells were identified and counted as CD4^+^CD25^+^FoxP3^+^ T cells within the CD4^+^ T-cell gate. To estimate the frequency of NY-ESO-1-specific and influenza-specific CD8^+^ T cells, PBMC from HLA-A2^+^ MM patients were first stained with either NY-ESO-1_157-165_ pentamers or influenza A_58-66_ pentamers for 15 minutes at room temperature and then with anti-CD8 and anti-CD19 mAb. After washings with PBS-BSA, the percentage of pentamer-positive cells was calculated after gating on CD19^-^ lymphoid cells on a two-color CD8 *vs*. pentamer plot, to exclude from the analysis all events that could be ascribed to non-specific binding of the pentamers to B cells.

### Cytokine assays

Cytokine production at the single-cell level was assessed with mAb directed against IL-2, IL-4, IL-10, IL-17A and IFN-γ. CD4^+^ cells were activated for 5 hours with 50 ng/ml PMA and 1 μg/ml ionomycin, in the presence of inhibitors of protein transport. For IL-10 detection, CD4^+^ cells were stimulated with LPS (1 μg/ml) for 24 hours, washed, fixed, permeabilized and then stained with pre-titrated amounts of cytokine-specific antibodies. Cytokine levels in patients’ serum or in culture supernatants were quantitated with commercially available ELISA. The limits of detection were as follows: 1 pg/ml IL-10; 7 pg/ml TGF-β1, 15.6 pg/ml IFN-γ, and 40 pg/ml HGF.

### Western blotting

Primary CD138^+^ cells or MM cell lines (6x10^5^) were centrifuged at 1,200 rpm for 10 minutes and cell pellets were lysed with RIPA buffer [150 mM NaCl, 1% NP-40, 0,5% sodium deoxycholate, 0,1% SDS, 50 mM Tris–HCl (pH=8), 1 mM PMSF, 1 mM EGTA, 50 mM NaF, 50 mM Na_3_VO_4_ and protease inhibitors (Roche, Milan, Italy)]. Cell lysates were incubated on ice for 20 minutes and clarified by centrifugation at 14,000 rpm for 20 minutes. Cell extracts obtained with RIPA buffer were boiled for 5 minutes at 95°C and analyzed by 12% SDS-PAGE. Samples were transferred onto nitrocellulose membrane (Bio-Rad, Milan, Italy). Blots were probed with primary antibodies at 1:1000 dillution, washed and developed with horseradish peroxidase-conjugated rabbit or mouse secondary antibodies (Bio-Rad), as appropriate. The bands were quantified densitometrically using the ImageJ software (National Institutes of Health, Bethesda, MD).

### Real-time quantitative PCR

Total RNA was obtained from cultured MM cells using the RNeasy plus kit (Qiagen, Milan, Italy) according to manufacturer’s instructions. Complementary DNA (cDNA) was prepared starting from 1 μg of total RNA using the iScript cDNA Synthesis Kit (Bio-Rad) according to the manufacturer’s instructions. Amplifications were carried out using specific primers (IDO1 gene: forward primer 5’→3’: GGGACACTTTGCTAAAGGCG; reverse primer 5’→3’: GTCTGATAGCTGGGGGTTGC) and the iQ SYBRGreen Supermix (Bio-Rad) in a final volume of 25 μL, starting with a 3-min template denaturation step at 95°C followed by 40 cycles of 15s at 95°C and 1 min at 60°C. β-actin was used as housekeeping gene (forward primer 5’→3’: GCCGACAGGATGCAGAAGGAG; reverse primer 5’→3’: CAGGATGGAGCCGCCGATC). Standard curves were generated using a serial dilution of the initial amount of control cDNA to determine the range of template concentrations, and showed a good linearity and efficiency for the different reactions. Melt curves of the reaction products were also generated to assess the specificity of the measured fluorescence. Samples were run in triplicate and the mean of threshold cycles (Ct) for each specimen was used to obtain the fold-change of gene expression level, using the following equation: fold change = 2 - (Ct), where Ct = Ct specific gene-Ct β-actin, and (Ct) = Ct specimen-Ct control. Calculations were made with the RelQuant Excel spreadsheet (Bio-Rad).

### Differentiation of Mo-DC

Circulating monocytes were isolated with CD14 Microbeads, as already published [33]. Monocytes were routinely >97% pure, as evaluated by FACS analysis. Cells were cultured for 7 days in GM-CSF/IL-4-containing medium, followed by 3 days of maturation in TNF-α-containing medium.

### Immunofluorescence analysis

Labeled cells were run through a FACS Canto® flow cytometer (BD Biosciences) with standard equipment. A minimum of 20,000 events was collected and acquired in list mode using the FACS Diva® software package (BD Biosciences).

### IDO1 activity

Tryptophan and KYN levels were measured with reverse-phase HPLC. Briefly, sample aliquots were deproteinized with 0.3 M HClO_4_. Supernatants were spiked with 50 μM 3-L-nitrotyrosine and analyzed using a ReproSil-Pur C18-AQ RP-HPLC column (Dr. Maisch GmbH, Ammerbuch-Entringen, Germany), using a double-pump HPLC apparatus from Jasco (Tokyo, Japan) equipped with spectrophotometric and fluorescence detectors. The chromatographic peaks were detected by recording UV absorbance at 360 nm and emission fluorescence at 366 nm, after excitation at 286 nm. The elution solvent was as follows: 2.7% CH_3_CN in 15 mM acetate buffer, pH 4.00 (both HPLC-grade; Fluka, Milan, Italy). The Borwin 1.5 and MS Excel software packages were used for instrument set-up and peak quantification. The concentration of components was calculated according to peak heights and was compared with both 3-nitro-L-tyrosine as internal standard and with reference curves constructed with L-tryptophan and KYN.

### Statistical methods

The approximation of data distribution to normality was tested preliminarily using statistics for kurtosis and symmetry. Data were presented as median and inter-quartile range and comparisons were performed with the Mann–Whitney U test for paired or unpaired data, or with the Kruskal-Wallis test with Bonferroni’s correction for multiple comparisons, as appropriate. The criterion for statistical significance was defined as a *p* value ≤ 0.05.

## Results

### IDO1 is functional and is mainly expressed by the malignant PC

**Figure 1:**
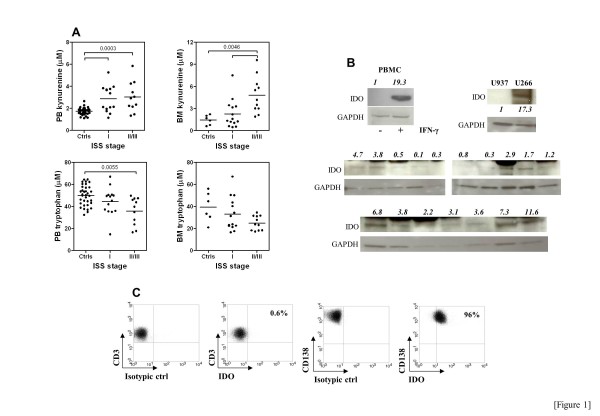
**IDO1 activity in patients with MM.** Panel **A**: KYN and tryptophan levels were measured with RP-HPLC both in patients with MM and in age-matched healthy controls (ctrls). Bars depict median values and inter-quartile ranges. Statistical comparisons were performed with the Kruskal-Wallis test for unpaired determinations with Dunns correction for multiple comparisons. Panel **B**: IDO protein was detected by Western blotting in malignant PC that were magnetically isolated with CD138 microbeads from the BM of 17 MM patients. The U937 monocytic leukemia cell line and U266 MM cell line were used as negative and positive control for IDO expression, respectively. Densitometry is indicated above each lane, relative to GAPDH. Panel **C**: BM mononuclear cells from 5 randomly selected MM patients were stained with an anti-human IDO mAb and then analyzed by flow cytometry. In this representative experiment, BM-resident T cells stained negatively for IDO, at variance with BM CD138^+^ PC. Markers were set according to the proper isotypic control.

In mice, most of the KYN formed through the action of IDO is pooled in both plasma and tissues [4]. Based on this observation, we measured tryptophan and KYN both in serum and in BM fluid collected from a cohort of 25 patients with newly diagnosed or relapsed MM, and in 7 patients with either SMM (n=4) or MGUS (n=3) as controls. In the overall population of patients with PC dyscrasia, KYN were higher both in the PB (2.92 μM/L, range 1.0-7.1) and in the BM (2.0 μM/L, range 0.45-9.57) as compared with age/sex-matched healthy controls (1.80 μM/L, range 1.11-2.65, and 1.5 μM/L, range 0.6-2.21, in PB *p*<0.0001] and BM *p*=0.0141], respectively), suggesting *in vivo* activation of IDO1. When MM patients were arbitrarily dichotomized based on serum KYN (lower or greater than the median in healthy controls, i.e., 1.8 μM), 19 out of 25 patients (75%) could be assigned to the KYN^hi^ MM group. KYN levels were also augmented in the PB and BM of patients with MGUS and SMM and no statistically significant differences in KYN release were recorded when comparing these patient groups (data not shown). As the composite results in Figure 1A clearly show, KYN were significantly more represented in the BM microenvironment of patients assigned to ISS stage II/III compared with those having less advanced disease. Furthermore, tryptophan concentrations both in PB and in BM were lower in MM patients with ISS stage II-III disease compared with MM patients with ISS stage I disease and with healthy controls (Figure 1A).

**Figure 2:**
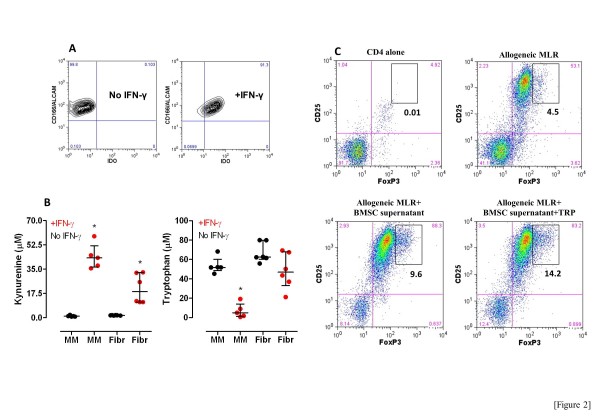
**IDO1 activity in MM BMSC.** Panel **A**: BMSC were generated as previously detailed [33] and were either left untreated or activated with 100 IU/ml IFN-γ for 24 hours. BMSC were fixed, permeabilized and stained with anti-IDO and anti-CD166 mAb. One representative experiment out of 5 with similar results is shown. Panel **B**: BMSC from 5 representative patients with MM and skin fibroblasts from 6 healthy subjects were either left untreated (black dots) or activated with 100 IU/ml IFN-γ for 24 hours (red dots) before measuring KYN and tryptophan level in culture supernatants. Comparisons between untreated and IFN-γ-stimulated samples were performed with the Wilcoxon matched-paired signed rank test. *p<0.01 compared with untreated samples. Panel **C**: Supernatants of BMSC cultures were transferred (20% v/v) to an MLR containing allogeneic naïve CD4^+^ T cells and third-party monocytes (at a fixed 1:3 ratio). IL-2 was provided to the cultures as a Treg growth factor. After 6 days, cells were harvested and labeled with anti-FoxP3 and anti-CD25 mAb to monitor the acquisition of a regulatory phenotype. A representative experiment out of 3 with similar results is shown. Markers were set according to the proper isotypic control (not shown). Rectangular gates were set to include CD4^+^CD25^hi^FoxP3^hi^ T cells.

Malignant PC were magnetically isolated in 17 MM patients with sufficient amounts of surplus diagnostic material, and the expression of IDO protein was investigated with Western blotting. As shown in Figure 1B, PC expressed readily detectable but varying levels of IDO in 12 out of 17 MM patients when compared with U937 monocytic leukemia cells used as negative control. In 5 randomly selected MM samples, we also assessed the expression of IDO protein by flow cytometry in purified CD138^+^ PC, which correlated with IDO levels as detected by WB. In all samples tested, CD3^+^ T cells stained negatively for IDO. One representative experiment is depicted in Figure 1C. We next asked whether cellular sources other than the malignant PC may account for IDO expression within the immune suppressive BM *milieu*. To accomplish this goal, we investigated IDO expression in patients’ BMSC that were either activated with exogenous IFN-γ for 24 hours or left untreated. Skin fibroblasts from healthy individuals were used as a control mesenchymal cell type. As shown in Figure 2A, IFN-γ challenge induced IDO protein in CD166^+^ BMSC from MM patients. KYN were not detected in unstimulated cultures (Figure 2B). Conversely, IFN-γ induced robust KYN production by MM BMSC that was associated with tryptophan consumption (Figure 2B). It should be noted that both KYN release and tryptophan breakdown were lower in supernatants of IFN-γ-stimulated skin fibroblasts compared with MM BMSC, suggesting that the latter cell type may be particularly sensitive to IFN-γ stimulation. The supernatants of IFN-γ-challenged MM BMSC were also transferred to an allogeneic MLR to determine whether IDO-driven tryptophan catabolites affect the acquisition of a Treg phenotype upon T-cell activation induced by alloantigens. As shown in Figure 2C, FoxP3^lo^ and FoxP3^hi^ cell populations were clearly distinguishable after flow cytometric analysis. The frequency of CD4^+^CD25^+^FoxP3^low^ T cells in the MLR cultures was significantly expanded irrespective of the presence of supernatants derived from MM BMSC and likely reflected T-cell activation in response to HLA-disparate stimulator cells. Interestingly, the provision of tryptophan-depleted and KYN-enriched supernatants from MM BMSC translated into a significant increase of CD4^+^CD25^+^FoxP3^hi^ Treg cells (Figure 2C). The addition of excess tryptophan to the MLR did not limit the expansion of either FoxP3^lo^ or FoxP3^hi^ Treg cells (Figure 2C), suggesting that the increase of Treg cells in the cultures was not merely due to tryptophan starvation but rather it resulted from increased KYN production. Collectively, these data indicated that, in the absence of pro-inflammatory stimuli such as IFN-γ, BMSC are unlikely to account for IDO1 expression/activity in patients with MM. In line with this assumption, we were unable to detect IFN-γ in the BM fluid of patients with MM (data not shown). A previous study also showed no constitutive IDO activity in BMSC from 5 patients with MM [36].

### IDO1 activity in MM correlates with the expansion of Treg cells and with the contraction of NY-ESO-1-specific CD8^+^ T cells

**Figure 3:**
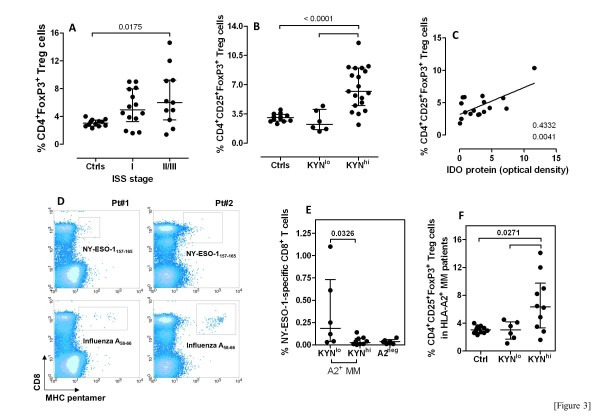
**IDO activity correlates with expanded Treg cells and with diminished NY-ESO-1-specific CD8^**+ **^T cells.** Panel **A**: The frequency of *bona fide* Treg cells in patients’ PB was estimated with flow cytometry, as already published [26]. Bars depict median values and inter-quartile ranges. Comparisons between MM patients and healthy controls were performed with Kruskal-Wallis test for unpaired determinations with Dunns correction for multiple comparisons. Panel **B**: Patients with MM were arbitrarily categorized into KYN^hi^ or KYN^lo^ if serum KYN were greater or lower than the median in healthy controls (1.8 μM), respectively. The frequency of Treg cells in MM patients and healthy controls was compared with the Kruskal-Wallis test for unpaired determinations. Bars illustrate median values and inter-quartile ranges. Panel **C**: The frequency of circulating Treg cells was correlated with IDO protein levels, as measured by Western blotting in purified PC from a subgroup of 17 patients with MM. r^2^=0.4332; *p*=0.0041. Panel **D**: The expression of HLA-A2 on PB leukocytes was preliminarily investigated by flow cytometry using an anti-HLA-A2 mAb in 22 MM patients whose malignant PC expressed NY-ESO-1 protein (Additional file 2A). Sixteen MM patients were HLA-A2^+^, whereas 6 were HLA-A2^-^. PBMC from the 16 HLA-A2^+^ MM patients were stained with NY-ESO-1 pentamers, as detailed in Materials and Methods. The frequency of influenza-specific CD8^+^ T cells was measured in each MM patient to rule out immune exhaustion. A representative experiment is shown. Panel **E**: The frequency of NY-ESO-1-specific CD8^+^ T cells was significantly lower in KYN^hi^ compared with KYN^lo^ MM patients. The very low percentage of NY-ESO-1-specific CD8^+^ T cells in the 6 HLA-A2^-^ MM patients reflects the background signal for pentamer staining. Panel **F**: The frequency of Treg cells in the 16 HLA-A2^+^ MM patients is shown with respect to IDO1 status. Bars depict median values and inter-quartile ranges.

We also asked whether IDO1 activity correlates with *in vivo* expansion of potentially immune-suppressive Treg cells. The gating strategy that we used to enumerate Treg cells in patients’ PB and BM is illustrated in Additional file 1. As shown in Figure 3A, the frequency of *bona fide* Treg cells in the PB was significantly higher in patients with higher stage MM compared with patients with ISS stage I disease and with healthy controls. The increase of Treg cells also within BMMC of patients with ISS stage III disease (median 6.2%, range 4.40-14.6) compared with stage II disease (median 9.3%, range 4.6-14.1), stage I disease (median 4.15%, range 1.0-7.6%) and with healthy donors (median 3.1%, range 2.3-4.0) suggested that Treg accumulation in the MM microenvironment may be one additional mechanism through which anti-myeloma immunity is restrained. The frequency of circulating Treg cells was superimposable when comparing patients having MGUS or SMM with those suffering from symptomatic MM (data not shown).

Interestingly, CD4^+^CD25^+^FoxP3^+^ Treg cells were more abundant in the PB of MM patients with heightened KYN levels (Figure 3B). The frequency of Treg cells also correlated with IDO protein levels, as determined by Western blotting (r^2^=0.4332, *p*=0.0041; Figure 3C). To get insights into the potential *in vivo* relevance of the above findings, we evaluated the frequency of myeloma-reactive, NY-ESO-1-specific CD8^+^ T cells in a subgroup of 16 HLA-A2^+^NY-ESO-1^+^ MM patients, 10 of whom could be classified as KYN^hi^ based on serum KYN (Figure 1A). The expression of NY-ESO-1 was tested with Western blotting in this subgroup of patients and is shown in Additional file 2*A*. Additional six HLA-A2^-^ MM patients served as negative control for pentamer staining. In the HLA-A2^+^ patients, the frequency of influenza-specific CD8^+^ T cells was measured together with that of NY-ESO-1-specific CD8^+^ T cells to rule out immune exhaustion (Figure 3D). Overall, compared with the KYN^lo^ MM patients, HLA-A2^+^KYN^hi^ MM patients had fewer NY-ESO-1-specific CD8^+^ T cells (Figure 3E), but higher frequencies of Treg cells (Figure 3F), indicating that, at least in a subset of MM patients, IDO1 activity may impact on anti-myeloma immunity through effects that include both the expansion of Treg cells and the contraction of myeloma-specific effector T cells. As already shown in the whole cohort of MM patients (Figure 3A), also in this subgroup of 10 KYN^hi^HLA-A2^+^ MM patients Treg cells were significantly more represented than in both KYN^lo^HLA-A2^+^ MM patients and healthy controls (Figure 3F).

### Myeloma cells lead to the differentiation of Treg cells *in vitro* through an IDO-dependent mechanism

**Figure 4:**
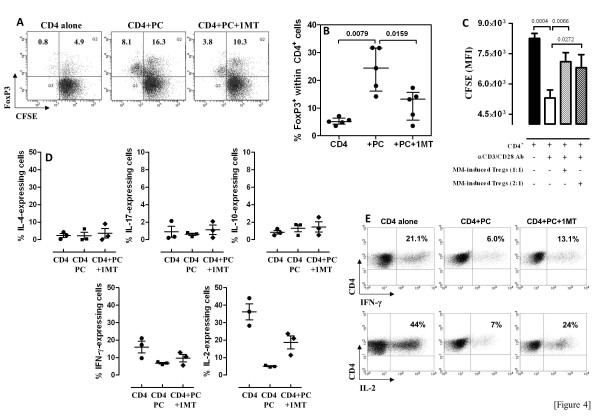
**IDO^**+ **^myeloma cells induce a Tr1/Th2 profile and suppress Th1 cytokines in allogeneic CD4^**+ **^T cells.** Panels **A** and **B**: Highly pure CD138^+^ PC from patients with MM were used for MLTC, as detailed in Materials and Methods. The allogeneic CD4^+^CD25^-^ T cells were pre-loaded with CFSE to monitor cell division. Exogenous IL-2 was provided to the MLTC at 100 IU/ml (final concentration). In selected wells, D,L-1MT was added at 200 μM (final concentration) to inhibit IDO activity. After 6 days in culture, cells were harvested and labeled with anti-FoxP3 and anti-CD25 mAb. Quadrant markers were set according to the proper isotypic control. A representative experiment is shown in panel **A**, whereas results are summarized in panel **B**. Panel **C**: CFSE dilution in responder CD4^+^CD25^-^ T cells was expressed in terms of mean fluorescence intensity of the dividing cell population (MFI). The bars depict the median and interquartile range recorded in 3 independent experiments performed in duplicate. The black column denotes T-cell cultures that were stimulated with anti-CD2+anti-CD3+anti-CD28 mAb in the absence of MM-induced Treg cells. Panel **D**: MM cells from KYN^hi^ patients were seeded in a MTLC as above detailed. After 6 days in culture, T cells were collected and used for intracellular cytokine staining. The percentage of IL-2, and IFN-γ-expressing T cells shown in panel **E** is representative of 3 independent experiments. Quadrant markers were set according to the proper isotypic control.

To test the hypothesis that IDO1-expressing myeloma cells sustain the amplification of Treg cells, allogeneic naïve CD4^+^ T cells were challenged *in vitro* with myeloma cells in a MTLC. The CD4^+^ T cells were pre-loaded with the fluorescent dye CFSE to track their proliferation. As evaluated through the combined staining with CFSE and anti-FoxP3 mAb (Figure 4A-B), IDO^+^ myeloma cells induced an expansion of the overall Treg population. These effects were inhibited, albeit not completely, by the provision of d,l-1MT to the co-cultures. To assess whether proliferating myeloma cells competed for tryptophan, leading to its relative depletion independent of IDO, MM cells were loaded with CFSE and cultured for 72 hours to track cell proliferation. As shown in Additional file 2*B*, MM cells had minimal proliferative activity when maintained with complete medium in the absence of exogenous growth factors. The MM-induced Treg cells were capable of constraining the proliferation of allogeneic CD4^+^ T cells induced by polyclonal stimulation with a cocktail of anti-CD2/CD3/CD28 mAb, indicating that they were functional (Figure 4C). The CD4^+^ T cells emerging from the MTLC were also activated with PMA and ionomycin and then assayed for intracellular cytokines assigned to the Th1, Th2 and Th17 lineage. A representative control experiment with commercially available Th1/Th2-polarized T cells is depicted in Additional file 3*A*. Figure 4D shows the results of 3 experiments where IDO-expressing MM cells inhibited the development of IFN-γ/IL-2-producing T cells *in vitro*. Conversely, IL-10, IL-17 and IL-4-expressing CD4^+^ T cells were unchanged after T-cell co-culture with MM cells. Interestingly, d,l-1MT partially reverted the diminished T-cell expression of IFN-γ and IL-2 in response to MM cells, and slightly increased IL-10 production at the single-cell level (Figure 4D). Figure 4E illustrates a representative experiment aimed at measuring IL-2 and IFN-γ production under the different culture conditions here established.

### IDO1 activity in MM patients correlates with the release of HGF but not other immune-suppressive cytokines

**Figure 5:**
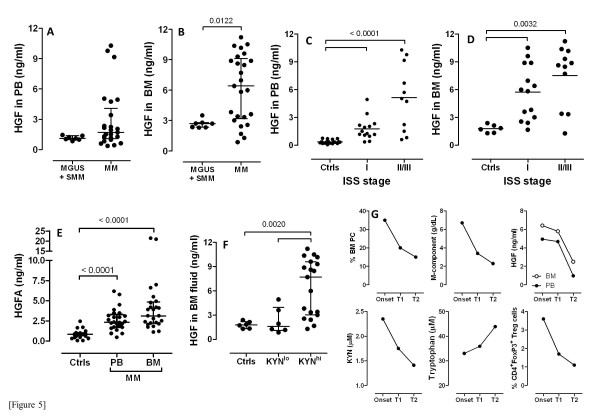
**HGF release correlates with disease burden and with IDO activity in patients with MM.** HGF levels were measured with ELISA both in PB and BM fluid of patients with MGUS, SMM and symptomatic MM at different ISS stages (panels **A**-**D**). HGF levels in patients and controls were compared with the Kruskal-Wallis test for unpaired determinations with Dunns correction for multiple comparisons. Bars depict median values and inter-quartile ranges. Panel **E**: HGFA in PB and BM fluid was measured in patients with MM using commercially available ELISA kits. Comparisons were performed with the Kruskal-Wallis test for unpaired determinations with Dunns correction for multiple comparisons. Panel **F**: HGF levels in the BM microenvironment were correlated with IDO activity, as expressed in terms of KYN release into the systemic circulation. When MM patients were dichotomized based on KYN levels (<1.8 μM or >1.8 μM), KYN^hi^ patients released significantly higher levels of HGF compared with KYN^lo^ patients. Comparisons were performed with the Kruskal-Wallis test for unpaired determinations with Dunns correction for multiple comparisons. Panel **G**: Disease burden, HGF release, KYN and tryptophan levels were measured in 1 patient with MM, both before (disease onset) and after treatment (T1 and T2 = 6 months and 1 year, respectively, after chemotherapy with bortezomib, melphalan and prednisone). The percentage of malignant PC as well as serum levels of M-component progressively declined, in correlation with serum and BM HGF, and with KYN.

**Figure 6:**
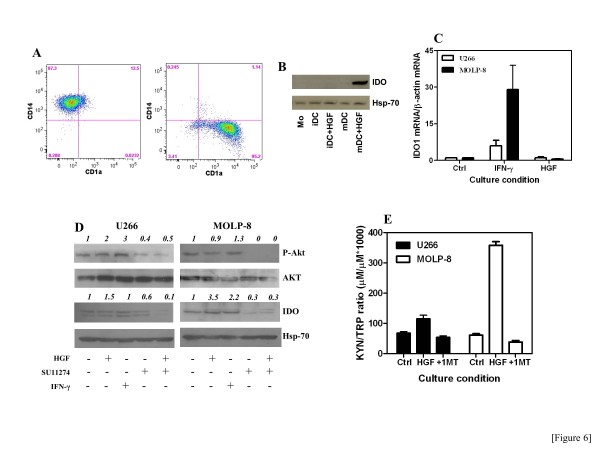
**HGF modulates IDO in MM cell lines.** Panel **A**: Mo-DC were differentiated from circulating monocytes of healthy donors with GM-CSF+IL-4 (7 days; immature Mo-DC or iDC) and then matured with TNF-α (2 additional days; mature DC or mDC), as already published [26]. Mature Mo-DC were routinely CD14^dim/neg^CD1a^+^, as determined by FACS analysis. Panel **B**: Mo-DC differentiated in the presence of exogenous HGF at 20 ng/ml and then matured with TNF-α up-regulated IDO protein. Panel **C**: U266 and MOLP-8 MM cells were stimulated with either IFN-γ or HGF for 24h. Messenger RNA levels for IDO were measured with quantitative RT-PCR relative to β-actin. Bars depict mean and SD recorded in 3 independent experiments. Panel **D**: U266 and MOLP-8 MM cells were stimulated with 20 ng/ml HGF for 24 hours. Cell lysates were immunoblotted with phosphorylation-specific antibodies against AKT (Ser473) [62]. SU11274, a selective MET inhibitor, was provided at 100 nM to the cultures. IFN-γ at 100 IU/ml was used as a prototypical inducer of IDO. Panel **E**: Tryptophan and KYN concentrations were measured in supernatants of U266 and MOLP-8 MM cells that were stimulated with 20 ng/ml HGF either in the presence or absence of 1MT. Results are representative of 3 independent experiments run in duplicate.

**Figure 7:**
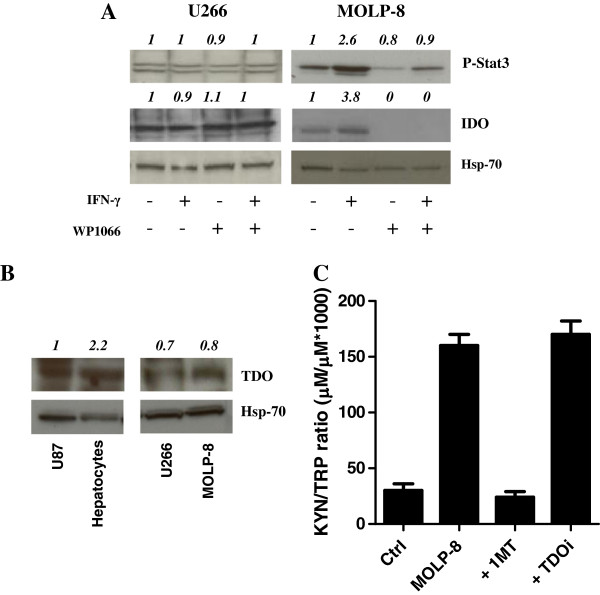
**STAT3 signaling and expression of TDO by MM cell lines.** Panel **A**: U266 and MOLP-8 MM cells were pre-treated with WP1066, a STAT3 inhibitor, and then activated with IFN-γ for 72h. Cell lysates were immunoblotted with phosphorylation-specific antibodies against STAT3 and with anti-human IDO antibodies. Densitometry is indicated above each lane, relative to Hsp-70. Panel **B**: The expression of TDO protein was measured in U266 and MOLP-8 MM cells, relative to U87 glioma cells and primary rat hepatocytes, used as positive control. Densitometry is indicated above each lane, relative to Hsp-70. Panel **C**: Tryptophan and KYN concentrations were measured in supernatants of MOLP-8 MM cells that were activated with 100 IU IFN-γ in the presence of either 1MT or 680C91, a selective TDO inhibitor (TDOi). Results are representative of 4 independent experiments run in duplicate.

We next focused our studies on candidate cytokine stimuli that may be responsible for IDO1 induction in MM. In this respect, both TGF-β and IL-10 are known to modulate IDO1 and may be expressed by the myeloma cells during progressive disease [37]. To this end, TGF-β and IL-10 levels were measured in a subgroup of 19 MM patients (Additional file 3*B*). Although both IL-10 and TGF-β were increased in some patients, both in the PB and BM, there was no correlation between IL-10/TGF-β release and serum KYN (data not shown). We subsequently quantitated HGF production both in PB and in BM fluid and showed that HGF was dramatically increased in patients with MM compared with those having MGUS or SMM, being particularly elevated in the immune-suppressive BM microenvironment (Figure 5A-B). Furthermore, HGF levels were higher in patients with ISS stage II/III disease compared with those having ISS stage I MM (Figure 5C-D), suggesting that the magnitude of HGF release reflects disease burden. Also, HGFA, whose presence is a pre-requisite for HGF activation *in vivo*[27], was higher in patients’ PB and BM fluid compared with healthy controls (Figure 5E). When attempting to correlate HGF levels with KYN production, we found that HGF was higher in the BM microenvironment of patients with KYN^hi^ MM compared with those having KYN^lo^ MM (Figure 5F). Also, serum HGF was not significantly different in the KYN^lo^ MM patients than in healthy controls. However, it should be emphasized that HGF levels overlapped in KYN^lo^ patients and in a subgroup of 8 out of 19 (42%) KYN^hi^ patients. Conversely, HGF levels were dramatically increased in the remaining 11 KYN^hi^ patients when compared with KYN^lo^ MM and with healthy controls. As shown in Figure 5G, disease burden, HGF release as well as KYN and tryptophan levels were evaluated serially in 1 patient with MM, both before (disease onset) and after treatment (T1 and T2 = 6 months and 1 year, respectively, after combination chemotherapy with bortezomib, melphalan and prednisone). The percentage of malignant PC and serum M-component progressively declined in correlation with lowered HGF and KYN release. Moreover, the percentage of Treg cells diminished after treatment compared with baseline. Collectively, these data suggest that high HGF production in MM may correlate with IDO1 activity and with disease burden. Finally, we aimed at establishing a mechanistic link between HGF stimulation and IDO expression by MM cells. Preliminarily, we confirmed our previous findings that HGF potently induces IDO protein in mature, CD14^-^CD1a^+^ Mo-DC (Figure 6A-B) [26]. Prompted by technical limitations in the collection of high numbers of primary PC from MM patients, we treated U266 and MOLP-8 MM cells with exogenous HGF and then analyzed IDO expression at mRNA and protein level, as well as KYN release in culture supernatants [38]. As shown in Figure 6C, mRNA levels for *IDO1* were unaffected by HGF, at variance with those detected in IFN-γ-stimulated MM cells. Treatment with HGF resulted in enhanced phosphorylation of AKT, as well as increased IDO1 expression and enzyme activity in MOLP-8 cells (Figure 6D). Of interest, 1MT reverted the heightened KYN/tryptophan ratio measured in supernatants of HGF-stimulated MOLP-8 cells, indicating that KYN production could be ascribed to IDO1 activity (Figure 6E). Figure 6D also illustrates that pre-treatment of U266 and MOLP-8 MM cells with SU11274, a MET inhibitor, antagonized both baseline and HGF-stimulated activation of AKT. The STAT3 pathway has been implicated in immune suppressive circuits [39]. In addition, HGF can induce STAT3 phosphorylation and STAT3, in turn, interacts with MET, both directly and indirectly [40]. As shown in Figure 7A, WP1066, a potent STAT3 inhibitor, down-regulated STAT3 phosphorylation as well as IDO protein expression in MOLP-8 MM cells, but not in U266 MM cells, suggesting that HGF may be implicated in IDO regulation through multiple signaling pathways, at least in some MM cases. Finally, we focused our studies on TDO expression and activity in MM cells. Figure 7B shows that TDO protein levels were low in MM cells compared with glioma cells and primary rat hepatocytes. At variance with 1MT, 680C91, a selective and potent TDO inhibitor, failed to revert the increased KYN/tryptophan ratio in supernatants of MM cells (Figure 7C), suggesting that KYN production by MM cells was mainly accounted for by IDO1 activity.

## Discussion

MM is a malignant PC disorder with a unique ability to subvert and/or escape immune responses. The interactions of MM cells with the BM microenvironment, either directly through inter-cellular adhesion or indirectly through the effects of growth factors, activate a variety of signaling cascades [22]. In this respect, HGF released by both MM cells and BMSC is rapidly emerging as a potential target for treatment both in MM and in B-cell lymphomas [41]. Importantly, MM should be amenable to therapies aimed at restoring anti-tumor immunity, since MM cells are sensitive to immune attack in the form of graft-versus-myeloma effect [42]. A large majority of human tumors, including prostate, pancreatic and colorectal carcinomas, express IDO in a constitutive manner [43]. IFN-γ is a prototypical IDO1-inducing cytokine in solid tumor cell lines, but not in cells of either lymphoid or myeloid origin, such as MM [44] and myeloid leukemia cells [45,46]. Our study evaluated the expression and function of IDO1 in human MM samples. In approximately 75% of unselected patients with symptomatic MM, IDO1 activation was reflected by the increase of serum KYN, the end-product of tryptophan breakdown by IDO. KYN levels were particularly elevated within the immune suppressive BM microenvironment of patients with ISS stage II/III myeloma disease. In the majority of MM patients, malignant PC cells were identified as a major source of IDO1 expression by Western blotting and flow cytometry. In agreement with previously published results [36], patient-derived BMSC were exquisitely sensitive to IFN-γ challenge, as shown by the robust KYN production and concomitant tryptophan depletion in culture supernatants. This observation has two potential implications. First, IFN-γ may not be the primary cytokine stimulus driving IDO expression in MM. Second, the IDO^-^ BMSC may start expressing IDO when exposed to an inflammatory *milieu* enriched in IFN-γ, implying that the immune resistance mechanisms centered on IDO could be exploited *in vivo* both by the myeloma cells and by microenvironmental cell types, such as BMSC. Notably, IFN-γ-challenged BMSC isolated from 5 patients with MM induced IDO-dependent tryptophan deprivation, leading to MM cell apoptosis and inhibited *in vitro* growth [36]. It is also conceivable that microenvironmental cell types other than malignant PC may be endowed with IDO activity. In this respect, BM-resident CD14^+^ monocytes and BDCA-4^+^ plasmacytoid DC from MM patients express readily detectable levels of IDO protein (Rutella S, unpublished observations, 2012). It remains to be determined whether other accessory cells partake in IDO-driven immune suppressive circuits in MM. In this respect, activation of the KYN pathway has been reported in patients with autoimmune diseases, such as rheumatoid arthritis, systemic lupus erythematosus and systemic sclerosis, likely as a result of IDO1 expression by non-hematopoietic cell types, such as fibroblasts, epithelial cells and vascular endothelial cells [47,48].

Conflicting reports have been published on the frequency of FoxP3-expressing Treg cells in patients with MGUS and MM, with studies showing either a decrease [49] or an increase of Treg cells [50]. In our cohort of MM patients, Treg cells were over-represented both in PB and BM, suggesting that Treg cells may mediate immune escape also in the BM microenvironment. Importantly, the *in vivo* expansion of *bona fide* Treg cells was more pronounced in patients with ISS stage II/III disease and correlated with both IDO1 expression in BM PC and enzyme activity, i.e., serum KYN levels. The NY-ESO-1 cancer/testis antigen is frequently over-expressed in MM, being a potential target for immunotherapy [51]. The percentage of NY-ESO-1-specific CD8^+^ T cells was significantly lower in KYN^hi^ MM patients and was negatively correlated with Treg-cell expansion, implying that IDO1 activity may affect the anti-myeloma immune response either directly or indirectly, through the Treg compartment. Co-cultures of allogeneic T cells with IDO-expressing MM cells indicated that Treg-cell expansion was mediated by IDO, as it could be inhibited, albeit not completely, by the provision of the racemic mixture d,l-1MT. We consistently observed a ~50% reduction of Treg conversion by the IDO^+^ myeloma cells, both in the presence of d,l-1MT and in the presence of the racemer l-1MT (data not shown). This may indicate that other mechanisms of immune subversion by MM, in addition to IDO1 expression, are operational or that 1MT-mediated inhibition of IDO1 was itself incomplete. The FoxP3-expressing Treg cells that emerged from the MM/T-cell co-cultures restrained allogeneic T-cell proliferation, suggesting that they were functional. *In vitro*, MM cells favored the differentiation of both FoxP3^lo^ and FoxP3^hi^ Treg cells. It has been reported that ‘naive’ Treg cells are characterized by their low levels of intracellular FoxP3 [52], whereas ‘effector’ Treg cells express a FoxP3^hi^ phenotype [53]. It is tempting to speculate that MM cells may deliver signals for initiating the expansion of both naïve Treg cells in a resting state and activated functionally differentiated effector Treg cells [54]. Furthermore, CD4^+^ T cells primed with IDO^+^ MM cells acquired a IFN-γ^dim^IL-2^dim^IL-4^+^IL-10^+^ cytokine secretion profile, consistent with type 1 Treg cells (Tr1)/Th2 cells. Other reports have also attributed to MM cell lines, such as U266 and RPMI-8226 cells, and to fresh MM cells the ability to down-regulate IFN-γ release by both un-activated and activated T lymphocytes [55]. Also, previous studies documented the capacity of myeloma cells to induce Tr1/Th2 cytokine production by T cells [56]. In our co-culture system, 1MT partially reverted the inhibition of Th1 cytokine production, suggesting that IDO-expressing MM cells may impinge both on Treg responses and on the Th1/Th2 balance [57].

Our interest in HGF as a candidate stimulus driving IDO1 expression in MM stems from studies underpinning the role of HGF in myeloma pathogenesis and from our previous report on the ability of HGF to induce IDO1 in human DC [26]. In our cohort of MM patients, HGF was over-expressed both in PB and BM, being particularly elevated in patients with ISS stage II/III MM. In addition, serial measurements of disease burden, HGF levels and KYN-to-tryptophan ratio in 1 MM patient showed that IDO1 activity decreased concomitantly with the reduction of both HGF release and PC infiltration in the BM microenvironment. It should be noted that, although HGF levels were collectively higher in KYN^hi^ compared with KYN^lo^ MM patients, a subgroup of 11 KYN^hi^ MM patients were characterized by remarkably heightened HGF levels. However, their clinical and biological characteristics were not significantly different from those of the other MM patients herein analyzed, including the prevalence of unfavorable cytogenetics, such as del(13q) and t(4;14) [58], the magnitude of BM infiltration with malignant PC at diagnosis or the ISS stage. The small number of patients, however, precluded any sensible conclusion on the impact of high IDO activity on overall survival, an issue that could be addressed by studies with larger cohorts of MM patients. Recently, a tumoral immune resistance mechanism centered on TDO has been described [5,14]. In our study, MM cell lines expressed low levels of TDO compared with glioma cell lines and with primary rat hepatocytes. The provision of a selective TDO inhibitor to MM cells did not affect KYN production nor tryptophan consumption, suggesting that TDO is unlikely to play a major role in tryptophan breakdown by MM cells. The HGF/MET pathway is an emerging target for the treatment of MM, B-cell lymphomas and solid tumors [59,60]. MET signaling is mainly mediated by the ERK-MAPK and PI3K-AKT pathways. However, several types of cooperation and crosstalk between MET and other signaling systems, such as TGF-β, WNT and others, have been unveiled in human cancer [61,62]. We confirmed that HGF is a powerful inducer of IDO in Mo-DC [26,63] and extended this observation to show that HGF may be implicated in the regulation of IDO expression also in MM cells. In support of this statement, the provision of a MET kinase inhibitor to both U266 and MOLP-8 MM cells translated into a remarkable down-regulation of IDO protein, in correlation with inhibited AKT phosphorylation. It should be pointed out that other signaling pathways, including those centered on STAT3 [64], may be operational in MM cells. This is supported by our observation that WP1066, a potent STAT3 inhibitor, abrogated IDO expression in MOLP-8 cells, either constitutive or induced by IFN-γ. Theoretically, the interaction between HGF and its receptor can be blocked by antagonists of either HGF or MET [65]. NK4, a truncated splice variant of HGF corresponding to the α chain of HGF, competitively antagonizes HGF and inhibits MM growth both *in vitro* and *in vivo*[66]. Intriguingly, NK4 in combination with DC vaccination inhibited the growth of experimental solid tumors, promoted tumor antigen-specific secretion of IFN-γ by CD8^+^ T cells and elicited MHC class I-restricted CTL activity against the parental tumor cells [67]. This study strongly suggests that HGF antagonism may break immune tolerance in tumor-bearing hosts.

## Conclusions

In conclusion, we provide evidence that IDO1 is expressed and has functional activity in the majority of patients with MM. *Ex vivo* studies point to HGF as one cytokine stimulus mediating IDO1 induction in MM. It remains to be determined whether targeting the HGF/MET axis in MM and other HGF-expressing cancers will interfere with the tumor-induced immune dysfunction through the inhibition of IDO activity.

## Competing interests

The authors have no competing interests.

## Authors’ contributions

GB, AM, DN, AP, LN, VF, RDC, MC, FL, GS and SR performed the laboratory work for this study. LDR, IM, AR, MG and AP recruited the patients. FL also provided intellectual input. SR conceived of the study, participated in its design and coordination and drafted the manuscript. All authors read and approved the final manuscript.

## Supplementary Material

Additional file 1 **Gating strategy for Treg enumeration.** The frequency of *bona fide* Treg cells was measured by multi-parameter flow cytometry in patients with MM. Representative experiments in MM patients with high and low KYN levels are shown. Markers were set according to the proper isotypic control. The percentage of cells staining positively for each antigen is indicated. Click here for file

Additional file 2 **Expression of NY-ESO-1 protein by MM cells and measurement of MM cell proliferation.** The expression of NY-ESO-1 was investigated by Western blotting relative to the GAPDH housekeeping protein (panel A). U937 monocytic leukemia cells and U266 MM cells served as negative and positive control for NY-ESO-1 expression, respectively. Densitometry was used to qualitatively detect changes in protein expression. Panel B: U266 MM cells were pre-loaded with CFSE and then cultured in complete medium for 72 hours. CFSE dilution was detected by flow cytometry. Unlabeled U266 MM cells are shown as control. Click here for file

Additional file 3 **Intracellular cytokine staining and measurement of TGF-β/IL-10 in MM patients.** Panel A: HiCK-1 and HiCK-2 Cytokine Positive Control Cells were used in preliminary experiments to optimize intracellular cytokine staining. Markers were set according to the proper isotypic controls. Panel B: PB and BM samples from 19 patients with MM were used to quantify TGF-β and IL-10 by ELISA. Click here for file
