# Supplementary material for: Indoleamine 2,3-dioxygenase 1 (IDO1) activity correlates with immune system abnormalities in multiple myeloma
Source: J Transl Med. 2012 Dec 11;10:247. doi: 10.1186/1479-5876-10-247 (PMC3543251; doi:10.1186/1479-5876-10-247)
Supplement: Supplementary file 3 [file 1479-5876-10-247-S3.doc]

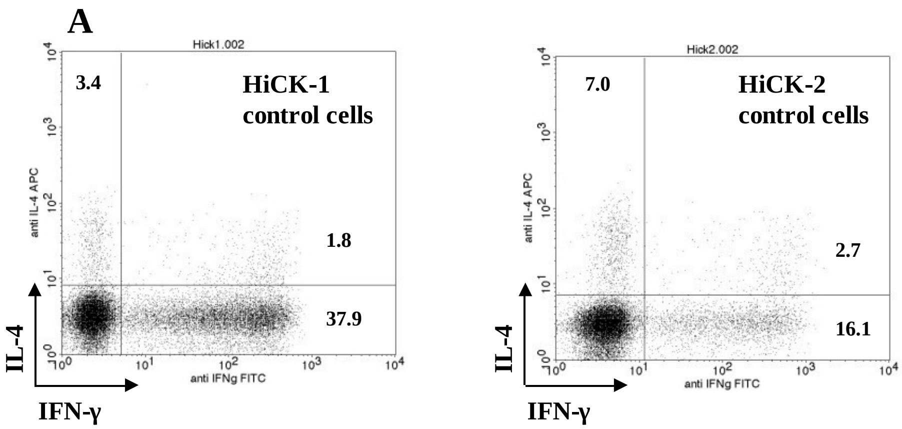

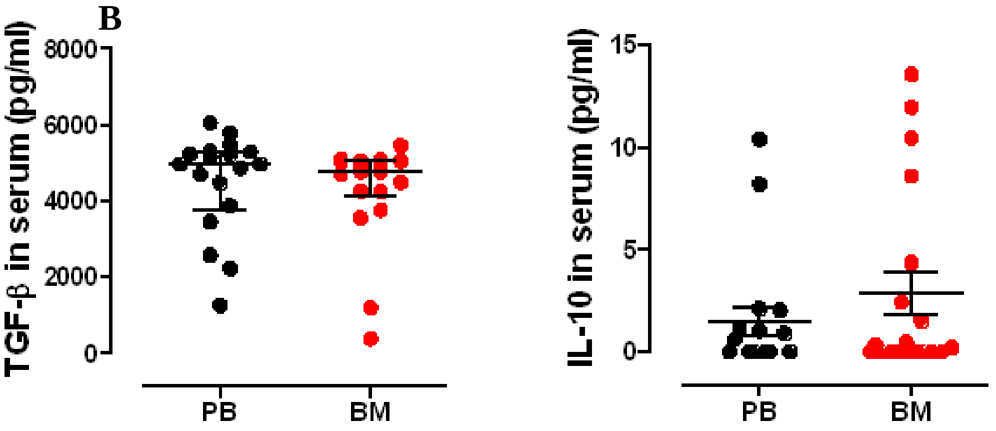

Additional file 3
